# Chiral Bis(Imidazolidine)Pyridine-Cu Complex-Catalyzed Enantioselective [3+2]-Cycloaddition of Azomethine Imineswith Propiolates

**DOI:** 10.3390/molecules17056170

**Published:** 2012-05-24

**Authors:** Takayoshi Arai, Yuta Ogino

**Affiliations:** Department of Chemistry, Graduate School of Science, Chiba University, 1-33, Yayoi, Inage, Chiba 263-8522, Japan

**Keywords:** pyrazolone, [3+2] cycloaddition, asymmetric synthesis, catalyst, chiral ligand

## Abstract

[3+2] Cycloaddition of azomethine imines with electron-deficient terminal alkynes was smoothly catalyzed by a chiral bis(imidazolidine)pyridine-CuOAc complex to give bicyclic pyrazolo[1,2-a]pyrazolone derivatives with up to 74% *ee*.

## 1. Introduction

**Figure 1:**
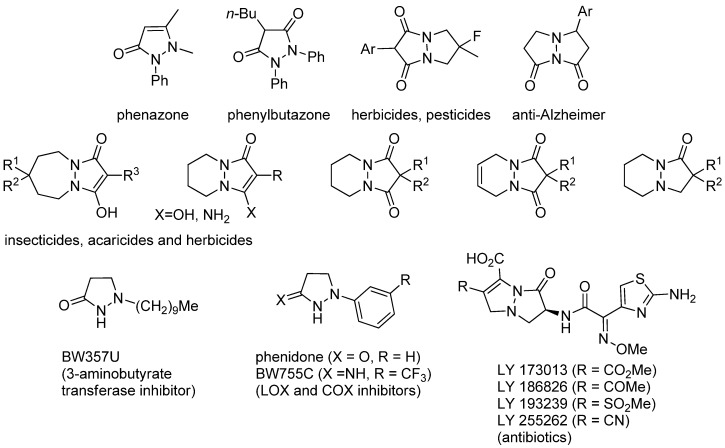
Representative examples of biologically active pyrazolone heterocycles.

Highly functionalized complex molecules are key tools for promoting biochemical research and developing pharmaceuticals, because the network of chiralities, the positions of heteroatoms, and the direction of lone-pairs in the molecules correlate strictly with their biological activities. The unique pyrazolone heterocycles, five-membered-ring lactams containing N-N conjunction, are examples of highly functionalized complex molecules, which have been used for the development of fine chemicals (e.g., dyes, textile, photography, and cosmetics) and the pharmaceuticals (Figure 1) [1,2,3]. Anti-inflammatory activity has been studied on phenylbutazones [4,5,6]. BW357U shows anorectic activity [7]. Inhibitory activities of lipoxygenase and cyclooxygenase are also reported on phenidone and BW755C [8,9]. More complex bicyclic pyrazolo[1,2-a]pyrazolone derivatives exhibit diverse bioactivities, and utilized as antibacterial agents, herbicides, pesticides, antitumor agents, calcitonin agonists, and potent drugs to relieve Alzheimer’s disease. LY173013 and LY186826 are the representative examples of antibacterial agents, which have been developed as the analogues of penicillin and cephalosporin antibiotics [10,11,12,13,14].

**Scheme 1:**
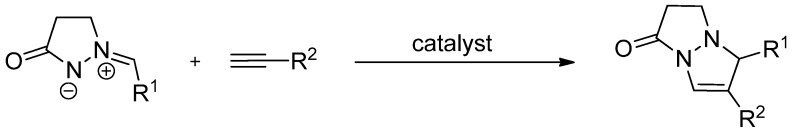
General scheme of [3+2] cycloaddition of azomethine imines.

For accessing the fascinating bicyclic pyrazolo[1,2-a]pyrazolone skeleton, the [3+2] cycloaddition of azomethine imines has apparent advantage for the straightforward construction of the core structure (Scheme 1) [15,16,17,18,19,20,21,22,23,24,25,26,27,28,29]. Due to the importance of these molecules on the biological study, the synthesis of these molecules in chiral form is highly demanded. In 2003, Fu *et al.* succeeded in the first catalytic asymmetric cyclization of azomethine imines and terminal alkynes using phosphaferrocene-oxazoline-CuI catalyst [30]. After the pioneering work of Fu’s group [30,31], several remarkable catalytic asymmetric [3+2] cyclizations using azomethine imines were reported by Sibi [32], Maruoka [33], Suga [34], and Chen [35,36].

**Scheme 2:**
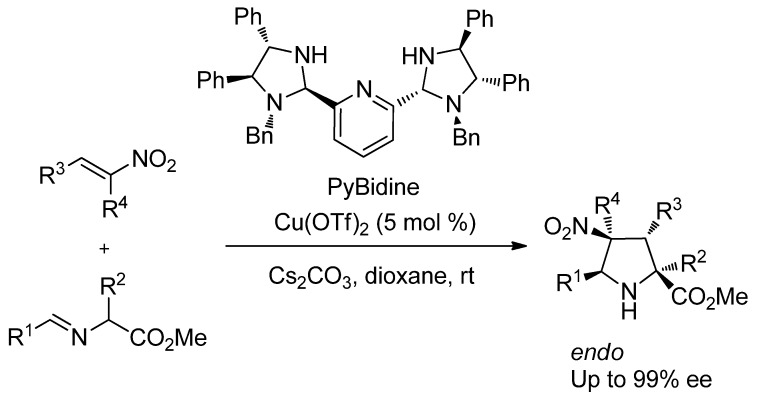
Asymmetric [3+2] cycloaddition catalyzed by PyBidine-Cu(OTf)_2_ complex.

In 2010 we succeeded in the development of bis(imidazolidine)pyridine, abbreviated as PyBidine, for the catalytic asymmetric [3+2] cycloaddition of iminoesters with nitroalkene (Scheme 2) [37]. We envisioned that the new chiral PyBidine ligand would have wide potential to promote various asymmetric reactions, and the [3+2] cycloaddition using azomethine imines was set to study for constructing the fascinating bicyclic pyrazolo[1,2-a]pyrazolones in this report. 

## 2. Results and Discussion

The study started with an exploration of the most appropriate metal salts for the [3+2] cycloaddition of azomethine imines with ethyl propiolate (Table 1).

**Table 1:** Catalyst activity of PyBidine-metal salt complex for [3+2] cycloaddition of azomethine imine with ethyl propiolate. 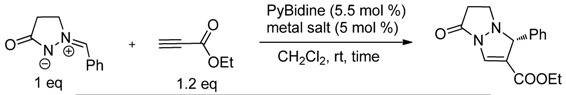

Entry	Metal salt	Time (h)	Yield (%)	*ee* (%)
1	CuI	3	99	44
2	CuBr	5	80	60
3	CuOAc	2	99	60
4	Cu(OAc)_2_	3	99	46
5	Cu(OTf)_2_	24	69	21
6	Co(OAc)_2_	31	trace	-
7	Ni(OAc)_2_	32	trace	-
8	Zn(OAc)_2_	50	trace	-

As reported in the former papers [30,31], Cu(I) salt showed good catalytic activity, and the PyBidine-CuI gave the product in 99% yield with 44% *ee* (rt, 3h). The PyBidine-CuOAc complex improved catalyst activity to give the product in 99% yield with 60% *ee* (entry 3). It was noteworthy that the Cu(II) salts also had catalyst activity, although the stereoselectivities of the product were reduced [27]. Because the other PyBidine-metal complex derived from the divalent acetate salts of the first row transition metals (Ni(OAc)_2_, Co(OAc)_2_, and Zn(OAc)_2_) gives traces of product, the copper element has unique and essential role for the [3+2] cycloaddition using azomethine imines. In all cases we examined, (*S*, *S*, *S*, *S*)-PyBidine-Cu catalyst gave the (*R*)-enriched product.

Next, the reaction conditions were optimized for the PyBidine-CuOAc catalyzed reaction (Table 2). The solvent effects were examined in the reaction using methyl propiolate. Various solvents (CHCl_3_, CH_2_Cl_2_, PhMe, THF, dioxane, EtOH) can be utilized to give the compound with good chemical yield, though the use of MeCN resulted in low yield. Among the solvents we examined, the reaction in CH_2_Cl_2_ gave the product with the best stereoselectivity (entry 2). 3,3-Dimethylpyrazolidinone also transformed to the bicyclic pyrazolo[1,2-a]pyrazolone in CH_2_Cl_2_ with 53% *ee* (entry 8). The azomethine imines prepared from substituted 4-iodo- or 3-chlorobenzaldehyde were examined in entries 11–14. A lower reaction temperature is helpful for improving the enantiomeric excesses in some cases (entries 9–10, and 13–14), though the reaction time was prolonged. To accelerate the reaction at low temperature, the addition of bases was also examined. The addition of DIPEA significantly accelerated the reaction to give the product with slightly improved enantioselectivity (entries 9 and 15), although the addition of pyridine or K_2_CO_3_ resulted in low stereoselectivity. For the ethyl propiolate, when the reaction was performed at −20 °C with DIPEA, the PyBidine-CuOAc gave the product with 74% *ee* (entry 19).

**Table 2:** PyBidine-CuOAc catalyzed [3+2] cycloaddition of azomethine imine with methyl propiolate ^a^. 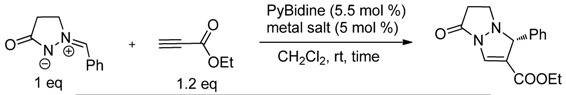

Entry	Ar	R^1^	R^2^	Base ^b^	Solvent	Temp (°C)	Time (h)	Yield (%)	*ee* (%)
1	Ph	H	Me	-	CHCl_3_	rt	24	66	55
2	Ph	H	Me	-	CH_2_Cl_2_	rt	4	95	67
3	Ph	H	Me	-	PhMe	rt	2.5	99	56
4	Ph	H	Me	-	MeCN	rt	27	39	52
5	Ph	H	Me	-	THF	rt	24	96	61
6	Ph	H	Me	-	dioxane	rt	24	95	35
7	Ph	H	Me	-	EtOH	rt	28	88	44
8	Ph	Me	Me	-	CH_2_Cl_2_	rt	24	67	53
9	Ph	H	Et	-	CH_2_Cl_2_	rt	2	99	60
10	Ph	H	Et	-	CH_2_Cl_2_	−10	45	97	65
11	4-IC_6_H_4_-	H	Et	-	CH_2_Cl_2_	rt	2	99	59
12	4-IC_6_H_4_-	H	Et	-	CH_2_Cl_2_	−10	23	58	54
13	3-ClC_6_H_4_-	H	Et	-	CH_2_Cl_2_	rt	3	88	47
14	3-ClC_6_H_4_-	H	Et	-	CH_2_Cl_2_	−10	23	62	62
15	Ph	H	Et	DIPEA	CH_2_Cl_2_	rt	1	99	70
16	Ph	H	Et	Pyridine	CH_2_Cl_2_	rt	1	99	40
17	Ph	H	Et	K_2_CO_3_	CH_2_Cl_2_	rt	16	90	32
18	Ph	H	Et	DIPEA	CH_2_Cl_2_	0	25	78	72
19	Ph	H	Et	DIPEA	CH_2_Cl_2_	−20	58	68	74

^a^ The absolute configurations of the products are provided by analogy with the products reported in ref. [30]; ^b^ 0.5 eq. of base were used to azomethine imine.

**Scheme 3:**
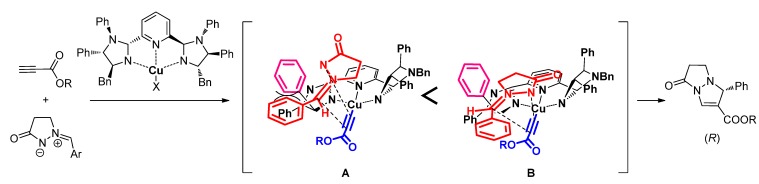
Plausible mechanism of asymmetric [3+2] cycloaddition catalyzed by PyBidine-CuOAc complex.

Although the enantiomeric excesses of the products are moderate, the reaction mechanism is assumed to be as summarized in Scheme 3. As proposed in former reports, the formation of copper acetylide would be a key for promoting the catalyst, though the Cu(II) salts also showed significant catalyst activity in our study. When the copper acetylide is generated on the (*S*,*S*,*S*,*S*)-PyBidine-Cu catalyst, the approach of (*Z*)-azomethine imine from an upper-side is considered on the reaction sphere. The approach of (*Z*)-azomethine imine to the Cu-acetylide would be assisted using the Lewis acidic apical cite of the Cu center. In a model **A**, a phenyl-ring of the PyBidine (emphasized by purple color) would have a steric repulsion with the aromatic ring of the (*Z*)-azomethine imine. To avoid the repulsion, the reaction would proceed *via* a model **B** to give the (*R*)-bicyclic pyrazolo[1,2-a]pyrazolone.

## 3. Experimental

### 3.1. General

Infrared (IR) spectra were recorded on a JASCO FT/IR-4100 Fourier transform infrared spectrophotometer. NMR spectra were recorded on a JEOL JNM-ECA500 or JEOL JNM-ECA400 spectrometer, operating at 500 MHz or 400 MHz for ^1^H-NMR and 125 MHz or 100 MHz for ^13^C-NMR. Chemical shifts in CDCl_3_ were reported downfield from TMS (=0 ppm) for ^1^H-NMR. For ^13^C-NMR, chemical shifts were reported downfield from TMS (=0 ppm) or in the scale relative to CHCl_3_ (77.00 ppm for ^13^C-NMR) as an internal reference. Optical rotations were measured on a JASCO P-1020 Polarimeter. The enantiomeric excess (*ee*) was determined by HPLC analysis. Column, DAICEL CHIRALPAK AD-H; mobile phase, hexane-*i*-PrOH; flow rate, 1.0 mL/min. General experimental details for synthesis of PyBidine ligand see reference in the manuscript [37]. Azomethine imine were synthesized according to known procedure [30].

### 3.2. General Procedure

PyBidine (0.0075 mmol) and CuOAc (0.00825 mmol) were added to a round flask containing a stir bar under Ar. Next CH_2_Cl_2_ (1 mL) was added to the flask and the mixture was stirred over 12 h. To the resulting solution, azomethine imine (0.15 mmol) were added, followed by ethyl propiolate (0.18 mmol) that was added dropwise at rt. After being stirred for an appropriate time, the reaction mixture was purified by silica gel column chromatography (Et_2_O/hexane = 2/1 to Et_2_O) to afford the corresponding cycloadduct. The enantiomeric excess of the products was determined by chiral stationary-phase HPLC analysis (Daicel CHIRALPAK AD-H, i-PrOH/hexane 20/80, flow rate 1.0 mL/min).

*(R)-Ethyl 5-oxo-1-phenyl-1,5,6,7-tetrahydropyrazolo[1,2-a]pyrazole-2-carboxylate*. Yellow oil; FTIR (neat) 3,403, 3,087, 3,063, 3,031, 2,980, 2,928, 2,853, 1,699, 1,602, 1,551, 1,499, 1,455, 1,433, 1,414, 1,388, 1,364, 1,321, 1,300, 1,251, 1,206, 1,173, 1,104, 1,077, 1,039, 1,027, 986, 914, 874, 842, 819, 765, 752, 725, 700, 663 cm^−1^; ^1^H-NMR (CDCl3) δ 7.54 (d, *J* = 0.5 Hz, 1H), 7.44–7.25 (m, 5H), 5.15 (d, *J* = 1.7 Hz, 1H), 4.15–3.95 (m, 2H) 3.41–3.36 (m, 1H) 3.08–3.00 (m, 1H) 2.95–2.83 (m, 1H), 2.79–2.70 (m, 1H), 1.13 (t, *J* = 7.2 Hz, 3H); ^13^C-NMR (CDCl_3_) δ 164.7, 163.4, 138.2, 128.5, 128.4, 128.3, 128.2, 118.5, 73.3, 60.4, 51.6, 35.7, 14.0; HRMS [ESI] Calcd. for C_15_H_16_N_2_O_3_Na^+^ [M+Na]^+^: 295.1053, found 295.1048; HPLC analysis: *t*_R_ 10.7 min (major, 87%) and 13.1 min (minor, 13%), detection at 330 nm]; [α]_D_^24^ −207 (c 1.01, CHCl_3_).

*(R)-Ethyl 1-(3-chlorophenyl)-5-oxo-1,5,6,7-tetrahydropyrazolo[1,2-a]pyrazole-2-carboxylate*. Yellow oil; FTIR (neat) 3,403, 3,086, 2,981, 2,927, 2,851, 1,697, 1,599, 1,474, 1,433, 1,387, 1,363, 1,319, 1,248, 1,198, 1,171, 1,105, 1,055, 1,024, 986, 934, 883, 842, 820, 785, 763, 735, 718, 693, 663 cm^−1^; ^1^H-NMR (CDCl3) δ 7.53 (s, 1H), 7.42 (s, 1H), 7.30 (d, *J* = 1.4 Hz, 3H), 5.12 (d, *J* = 1.8 Hz, 1H), 4.15–3.95 (m, 2H), 3.50–3.45 (m, 1H), 3.09–3.02 (m, 1H), 2.98–2.89 (m, 1H), 2.78–2.72 (m, 1H), 1.16 (t, *J* =7.2 Hz, 3H); ^13^C-NMR (CDCl_3_) δ 164.7, 163.2, 140.9, 134.4, 129.6, 128.7, 128.5, 128.3, 126.5, 73.0, 60.5, 52.1, 35.5, 14.0; HRMS [ESI] calcd for C_15_H_16_N_2_O_3_Cl^+^ [M+H]^+^: 307.0844, found 307.0839; HPLC analysis: *t*_R_ 9.1 min (major, 81%) and 11.1 min (minor, 19%), detection at 330 nm]; [α]_D_^24^ −362 (*c* 1.00, CHCl_3_).

*(R)-Ethyl 1-(4-iodophenyl)-5-oxo-1,5,6,7-tetrahydropyrazolo[1,2-a]pyrazole-2-carboxylate*. Yellow oil; FTIR (neat) 3,403, 3,084, 2,981, 2,925, 2,851, 2,309, 1,696, 1,604, 1,509, 1,434, 1,388, 1,364, 1,319, 1,250, 1,171, 1,105, 1,026, 986, 921, 849, 796, 758, 732, 664 cm^−1^; ^1^H-NMR (CDCl_3_) δ 7.53–7.52 (m, 1H), 7.40–7.36 (m, 2H), 7.09–7.03 (m, 2H), 5.14 (d, *J* = 1.6 Hz, 1H), 4.13–4.01 (m, 2H) 3.46–3.41 (m, 1H), 3.08–3.01 (m, 1H), 2.97–2.90 (m, 1H), 2.79–2.72 (m, 1H), 1.15 (t, *J* = 7.2, 3H); ^13^C-NMR (CDCl_3_) δ 164.6, 163.3, 134.4, 129.86, 129.79, 128.5, 118.1, 115.5, 115.3, 72.8, 60.4, 51.8, 35.6, 14.0; HPLC analysis: *t*_R_ 9.4 min (major, 77%) and 13.2 min (minor, 23%), detection at 330 nm]; [α]_D_^24^ −290 (*c* 1.00, CHCl_3_).

*(R)-Methyl 5-oxo-1-phenyl-1,5,6,7-tetrahydropyrazolo[1,2-a]pyrazole-2-carboxylate*. Yellow solid; FTIR (neat) 3,373, 3,083, 2,921, 2,862, 1,995, 1,714, 1,604, 1,496, 1,456, 1,407, 1,305, 1,246, 1,210, 1,167, 1,105, 958, 910, 836, 808, 771, 753, 735, 700 cm^−1^; ^1^H-NMR (CDCl3) δ 7.53 (brs, 1H), 7.38–7.31 (m, 5H), 5.17 (d, *J* = 1.6 Hz, 1H), 3.62 (s, 3H), 3.41–3.36 (m, 1H), 3.08–3.01 (m, 1H), 2.94–2.85 (m, 1H), 2.79–2.72 (m, 1H);^ 13^C-NMR (CDCl_3_) δ 164.7, 163.8, 138.1, 128.6, 128.5, 128.2, 117.9, 73.1, 51.5, 35.7; HRMS [ESI] Calcd for C_14_H_15_N_2_O_3_^+^ [M+H]^+^: 259.1077, Found 259.1073; HPLC analysis: *t*_R_ 11.3 min (major, 84%) and 12.7 min (minor, 16%), detection at 330 nm]; [α]_D_^24^ −296 (*c* 1.00, CHCl_3_).

*(R)-Methyl 7,7-dimethyl-5-oxo-1-phenyl-1,5,6,7-tetrahydropyrazolo[1,2-a]pyrazole-2-carboxylate*. Yellow solid; FTIR (neat) 3,414, 3,076, 3,030, 2,950, 1,695, 1,600, 1,495, 1,455, 1,385, 1,322, 1,256, 1,227, 1,120, 1,102, 1,042, 1,028, 1,006, 962, 913, 888, 847, 803, 755, 715, 698 cm^−1^; ^1^H-NMR (CDCl_3_) δ 7.52 (d, *J* = 1.6 Hz, 1H), 7.46–7.26 (m, 5H), 5.46 (brs, 1H), 3.62 (s, 3H), 2.87 (d, *J* = 15.6 Hz, 1H), 2.40 (d, *J* = 15.9 Hz, 1H), 1.24 (s, 3H), 1.15 (s, 1H); ^13^C-NMR (CDCl_3_) δ 166.6, 164.1, 142.0, 129.4, 128.4, 127.9, 127.8, 116.8, 64.51, 64.46, 51.4, 49.3, 24.9, 18.9; HPLC analysis (*i*-PrOH/hexane 10/90, flow rate 1.1 mL/min: *t*_R_ 9.0 min (minor, 24%) and 9.8 min (major, 76%), detection at 330 nm.

## 4. Conclusions

In conclusion, we have examined the application of PyBidine-Cu(OAc) complex for the [3+2] cycloaddition of azomethine ylide and propiolate for the construction the bicyclic pyrazolo[1,2-a]pyrazolone skeletons with 74% *ee*. This shows the broad utility of the PyBidine ligand for various metal-catalyzed reactions. In addition to a detailed study on the reaction mechanism, the extensive application to other catalytic reactions based on the unique functions of PyBidine is in progress.
